# The Situational Version of the Brief COPE: Dimensionality and Relationships With Goal-Related Variables

**DOI:** 10.5964/ejop.v11i2.935

**Published:** 2015-05-29

**Authors:** Dario Monzani, Patrizia Steca, Andrea Greco, Marco D’Addario, Erika Cappelletti, Luca Pancani

**Affiliations:** a Department of Psychology, University of Milano – Bicocca, Milan, Italy; University of Western Ontario, London, Canada

**Keywords:** coping, Brief COPE, confirmatory factor analysis, self-regulation theory, personal goals

## Abstract

This study is aimed at investigating the dimensionality of the situational version of the Brief COPE, a questionnaire that is frequently used to assess a broad range of coping responses to specific difficulties, by comparing five different factor models highlighted in previous studies. It also aimed at exploring the relationships among coping responses, personal goal commitment and progress. The study involved 606 adults (male = 289) ranging in age from 19 to 71. Using confirmatory factor analysis, we compared five models and assessed relationships of coping responses with goal commitment and progress. The results confirmed the theoretical factor structure of the situational Brief COPE. All the 14 dimensions showed acceptable reliability and relationships with goal commitment and progress, attesting the reliability and usefulness of this measure to evaluate coping responses to specific events.

## Coping

Coping is defined as the process of executing a response to a stressor, where stress is viewed as the experience of encountering relevant difficulties in one’s goal-related efforts (Lazarus, 1966). Within the framework of self-regulation theory, Carver and Connor-Smith (2010) explicitly highlighted the relationships among stress, coping, and goals. These authors affirmed that stress is closely linked to the pursuit of goals or to the avoidance of threats. Stress exists when an individual perceives the achievement of a desired goal as impossible or detects possible future punishments. People’s coping responses to threats and stressors are key determinants of their psychological adjustment and well-being. Thus, it is important to reliably and validly assess individual coping thoughts or actions. The Brief COPE (Carver, 1997) is one of the most frequently used self-report measures of coping responses. However, there is a lack of empirical evidence regarding its psychometric properties. The main aim of this study was to examine the dimensionality of the Brief COPE by comparing five different factor models highlighted by previous studies. Moreover, given the theoretical link between coping and goals, second aim was to evaluate the associations between coping responses and goal-related variables of commitment and progress.

Coping is a very broad concept and several distinctions have been made within this broad domain. One important distinction in the study of coping is the difference between situational coping responses and dispositional coping styles. The former correspond to the ways people react and cope with specific difficulties and stressful circumstances, whereas the latter are defined as tendencies to use specific coping reactions to a greater or lesser degree under stress (Carver & Scheier, 1994). Empirical evidence suggests that the adoption of situational coping responses is influenced by several different aspects, such as socioeconomic status (e.g., McIlvane, 2007), the dispositional coping style (Carver & Scheier, 1994), dispositional optimism (see Solberg Nes & Segerstrom, 2006 for a review), causal attributions (see Roesch & Weiner, 2001 for a review), and the controllability of stressors (Clarke, 2006; Coyle & Vera, 2013; Landis et al., 2007).

People generally exhibit high variability in their responses to threats and stressors, and several distinctions have been made between different types of coping. Initially, Folkman and Lazarus (1980) distinguished between *problem-focused coping* (i.e., strategies aimed at solving and actively responding to stressful situations) and *emotion-focused coping* (i.e., strategies to manage or reduce emotions and feelings that are embedded within stressful situations). Carver, Scheier, and Weintraub (1989) further distinguished between *approach coping* (i.e., strategies aimed at dealing actively with the stressor or related emotions) and *avoidance coping* (i.e., strategies aimed at avoiding stressful situations). The distinction between approach vs. avoidance coping is independent from the distinction between problem-focused vs. emotion-focused coping (Solberg Nes & Segerstrom, 2006). Therefore, people may cope with a stressor’s emotional consequences by either approaching or avoiding them, and people may cope with stressors themselves by actively and directly approaching or avoiding problems.

The relationship between coping strategies and personal goal pursuit is particularly evident when considering the effectiveness and adaptiveness of the coping responses adopted to face stressors. Concretely, a coping response is generally considered adaptive when it leads to a greater likelihood of making more progress and attaining desired goals (Carver & Scheier, 1998; Lazarus, 1991; Wrosch, Scheier, Carver, & Schulz, 2003). Similarly, ineffective coping responses are more likely to interfere with goal-directed behaviors and subsequent levels of performance (Weiss & Cropanzano, 1996). The link between problem-focused, approach coping responses and goal-related behavior is deeply rooted in the self-regulation theory proposed by Carver and Scheier (1998). Specifically, these types of coping strategies aim to actively manage the stressor itself. People who adopt both of these coping responses keep on be committed to and strive for their goals; consequently, they are more likely to report higher rates of progress or to attain their goals. Thus, approach coping responses are more likely to facilitate goal attainment and the experience of positive affect (Mackay, Charles, Kemp, & Heckhausen, 2011). On the contrary, avoidance coping leads to at least temporary disengagement and abandonment of goal-related behaviors. Consequently, it is more likely to impede and interfere with actual goal progress. Empirical evidence has underlined the important relationship between coping strategies and goal achievement in different life circumstances. In particular, the influence of approach coping and problem-focused responses on levels of goal progress has been evaluated and demonstrated in the education (Endler, Kantor, & Parker, 1994; Struthers, Perry, & Menec, 2000) and sport domains (Amiot, Gaudreau, & Blanchard, 2004; Gaudreau & Blondin, 2004).

## The Situational Version of The Brief COPE

Several self-report measures of coping responses have been developed and are currently available, including the Ways of Coping Questionnaire (WCQ; Folkman & Lazarus, 1988), the Coping Orientation to Problems Experienced (COPE; Carver, Scheier, & Weintraub, 1989), the Multidimensional Coping Inventory (MCI, Endler & Parker, 1990), the Coping Inventory for Stressful Situations (CISS; Endler & Parker, 1994), and the Coping Responses Inventory-Youth (CRI-Youth; Moos, 1993). All of these measures validly and reliably assess both approach and avoidance coping responses and both problem- and emotion-focused strategies. However, one possible drawback of these scales is their relatively extended length, ranging from 48 to 66 items, which may limit their usefulness in long research protocols and in clinical settings.

To overcome this potential shortcoming, Carver (1997) developed the Brief COPE, an abridged version of the COPE. The Brief COPE measures 14 theoretically identified coping responses: Self-distraction, Active coping, Denial, Substance use, Use of emotional support, Use of instrumental support, Behavioral disengagement, Venting, Positive reframing, Planning, Humor, Acceptance, Religion, and Self-blame. It represents a way to rapidly measure coping responses because it is a short 28-item self-report questionnaire with two items for each of the measured coping strategies. The list below reports items of the Brief COPE.

**The Situational Version of the Brief COPE (Carver, 1997; retrieved from www.psy.miami.edu/faculty/ccarver/sclBrCOPE.html)**
1. I've been turning to work or other activities to take my mind off things.
2. I've been concentrating my efforts on doing something about the situation I'm in.
3. I've been saying to myself "this isn't real".
4. I've been using alcohol or other drugs to make myself feel better.
5. I've been getting emotional support from others.
6. I've been giving up trying to deal with it.
7. I've been taking action to try to make the situation better.
8. I've been refusing to believe that it has happened.
9. I've been saying things to let my unpleasant feelings escape.
10. I’ve been getting help and advice from other people.
11. I've been using alcohol or other drugs to help me get through it.
12. I've been trying to see it in a different light, to make it seem more positive.
13. I’ve been criticizing myself.
14. I've been trying to come up with a strategy about what to do.
15. I've been getting comfort and understanding from someone.
16. I've been giving up the attempt to cope.
17. I've been looking for something good in what is happening.
18. I've been making jokes about it.
19. I've been doing something to think about it less, such as going to movies, watching TV, reading, daydreaming, sleeping, or shopping.
20. I've been accepting the reality of the fact that it has happened.
21. I've been expressing my negative feelings.
22. I've been trying to find comfort in my religion or spiritual beliefs.
23. I’ve been trying to get advice or help from other people about what to do.
24. I've been learning to live with it.
25. I've been thinking hard about what steps to take.
26. I’ve been blaming myself for things that happened.
27. I've been praying or meditating.
28. I've been making fun of the situation.

Each item presents a coping thought or action that individuals may adopt under stress or in difficult situations. For each item, respondents indicate whether they have used the coping response on a four-point Likert scale (1 = I haven't been doing this at all; 2 = I've been doing this a little bit; 3 = I've been doing this a medium amount; 4 = I've been doing this a lot). The items of the original version of the Brief COPE were in a format that was situational and retrospective, allowing for the assessment of situational coping responses to specific stressors. Carver’s instructions of the Brief COPE adopt a procedure similar to the one developed by Folkman and Lazarus (1980). Specifically, the Brief COPE asks participants to bring to mind a relevant stressor they encountered in the recent past and to indicate how they coped with it. Accordingly, the items of the situational version are expressed in the present perfect tense.

The Brief COPE has recently been used in empirical research evaluating the role of coping in facing different types of stressors, such as heart failure (Bean, Gibson, Flattery, Duncan, & Hess, 2009; Carels et al., 2004; Klein, Turvey, & Pies, 2007; Paukert, LeMaire, & Cully, 2009), HIV disease (Sanjuán, Molero, Fuster, & Nouvilas, 2013), terrorism (Stein et al., 2013), and caregiving for a family member with mental illness (Wrosch, Amir, & Miller, 2011). However, the Brief COPE’s theoretically based 14-factor structure (i.e., Model A) has received little empirical support. Specifically, the 14-factor structure has been confirmed only by Muller and Spitz (2003), who performed a confirmatory factor analysis (CFA) on responses to the French situational version of the Brief COPE provided by only 178 university students.

The dimensionality of the situational Brief COPE has been analyzed in three further studies. However, none of these studies performed a CFA to assess the 14-factor structure proposed by Carver (1997). Specifically, two (i.e., Carver, 1997; Miyazaki, Bodenhorn, Zalaquett, & Ng, 2008) of the three studies performed an exploratory factor analysis (EFA), whereas the fourth study (Knoll, Rieckmann, & Schwarzer, 2005) performed a second-order CFA to further summarize the 14 dimensions into four higher-order factors. Carver (1997) was the first to propose a factor structure different from the 14-factor model. He identified, through an EFA, only nine factors with eigenvalues greater than one (i.e., Model B). Five a priori scales formed distinct factors: Substance use, Religion, Humor, Behavioral disengagement, and Acceptance. Another factor was formed by Active coping, Planning, and Positive reframing items. Similarly, three other factors were formed by items from two distinct a priori scales. The Use of emotional support and Use of instrumental support items loaded together on a single factor. Another factor was formed by the Venting and Self-distraction items, and items from the Denial and Self-blame dimensions loaded together on another factor.

Knoll et al. (2005) assessed the higher-order dimensionality of the situational version of the German adaptation of the Brief COPE. The sample consisted of only 110 patients subjected to cataract surgery. The participants were asked to think about their actions and thoughts regarding the upcoming surgery and to respond to the items of the Brief COPE. Because of their low reliability, three subscales (i.e., Self-distraction, Substance use, and Behavioral disengagement) were excluded from the analysis. Thus, authors evaluated a first-order model (i.e., Model C) with only 11 dimensions of the 14 dimensions of the Brief COPE. Following suggestions by Carver et al. (1989) and considering previous results on the dimensionality of the Cope (Carver et al., 1989) and correlations among the dimensions of coping response, the authors performed a second-order CFA to further summarize these dimensions into four higher-order factors (i.e., Model D): Focus on the Positive (Acceptance, Positive reframing, and Humor), Support Coping (Use of instrumental Support, Use of emotional Support, and Religion), Active Coping (Active coping and Planning), and Evasive Coping (Self-blame, Denial, and Venting). This tested model yielded acceptable fit statistics and gave support to their hypothesized factor structure of four second-order dimensions of coping responses.

Lastly, Miyazaki et al. (2008) explored the dimensionality of the situational version of the Brief COPE in a sample of 555 international students in the United States. They performed an ordinal EFA that extracted seven factors (i.e., Model E). Only three dimensions (i.e., Substance use, Humor, and Religion) perfectly resembled the relative original domains, whereas the remaining four dimensions, labeled Positive coping, Self-blame, Support seeking, and Denial, were formed by items from different original domains.

## Purpose of the Study

The inconsistent empirical evidence reviewed above may raise questions about the Brief COPE true dimensionality and thus may limit its usefulness in research and applied settings. Specifically, the differences in the use of the Brief COPE and in the computing of its dimensions among studies may limit the comparability and validity of their findings. Moreover, previous studies evaluated Brief COPE’s factor structure by performing analyses on homogeneous and small samples. These small sample sizes may bias the validity of the results, whereas homogeneity may limit their generalizability. Consequently, the main aim of the present study is to shed light on the factor structure of the situational version of the Brief COPE. The identification of the best factor structure of this self-report measure is essential to properly assess coping strategies in empirical research and clinical practice. Thus, the study examined the dimensionality of the questionnaire by considering the previously proposed factor structures. Using CFA, we compared five models: Model A, Model B, Model C, Model D, and Model E. Table 1 summarizes these five models and their respective authors. In contrast to previous studies, we collected data on the Brief COPE from a large and heterogeneous sample ranging in age from 19 to 71.

**Table 1:** The Five Compared Models, Descriptions, and References

Models	Description	Reference
Model A	Theoretically based 14-factor structure	Carver, 1997; Muller & Spitz, 2003
Model B	EFA-based 9-factor structure	Carver, 1997
Model C	First-order 11-factor structure	Knoll et al., 2005
Model D	Second-order 4-factor structure	Knoll et al., 2005
Model E	7-factor structure	Miyazaki et al., 2008

Within the framework of the self-regulation theory and considering previous empirical evidence on coping responses and goal-related behavior, a secondary aim was to evaluate the relationships between coping responses and goal-related aspects of commitment and perceived progress. Specifically, we assessed coping responses to a specific personal goal-related difficulty and analyzed the relationships between identified coping responses and personal goal commitment and progress. Because of the cross-sectional design of this study, we considered goal progress as a proxy of goal performance but not goal attainment per se. We hypothesized the following:

- Goal commitment would be positively related to coping responses aimed at dealing actively with the stressors or related emotions and negatively correlated with coping responses aimed at avoiding stressful situations;
- Goal progress would be positively related to strategies aimed at dealing actively, solving and actively responding to stressful situations, and negatively correlated to responses aimed at avoiding stressors.

## Method

### Participants

A convenience sample of 606 participants was recruited. The participants were part of a broader research project designed to evaluate the relations among individual differences, personal goals, and well-being. The sample comprised 289 male and 317 female young adults from Northern Italy. The mean age was 30.93 (*SD* = 12.51) years (range: 19-71 years). Regarding occupational status, 41.5% of the participants were white-collar workers, 23.7% were university students, 12.8% were freelance workers, 9.9% were blue-collar workers, 4.2% were retired, 4.2% were homemakers, and 3.7% were unemployed. Considering educational levels, most participants (59.2%) possessed a high-school diploma, and 27.1% possessed a university degree. The remaining 13.7% had a lower educational level. Lastly, 46.9% of the participants were single, 48.9% were married and lived with their partner, 3.9% were divorced, and 0.3% were widowed.

### Measures and Procedure

The research received approval from the Ethics Committee of the University of Milan - Bicocca. Using the snowball sampling method, the participants were recruited by trained students enrolled in an undergraduate psychology course. The target population was individuals aged 18 or older. The participants were asked to carefully read and sign the informed consent form and to individually complete the questionnaire and then to return the items to the principal investigator. The participants did not receive any incentive for participation.

The situational Italian version of the Brief COPE was developed using the method of forward-backward translation (Brislin, 1970). The first author completed the translation of items of the Brief COPE. A second researcher blind to the content of the original English words performed the back translation. Both translators are bilingual but native Italian speaker. The results of the back translation were virtually identical to the original English version.

To administer the Brief COPE, we adopted a slightly modified version of the guidelines adopted by Folkman and Lazarus (1980). Our initial instructions asked participants to declare the most important goal they wished to achieve. The participants were then asked to describe a difficulty they faced in pursuing this goal during the last month. Then, the participants answered the 28 items of the Brief COPE to indicate the way in which they had coped with this difficulty. The response format was a four-point Likert scale ranging from “I haven’t been doing this at all” (1) to “I’ve been doing this a lot” (4).

To assess relationships of coping responses with goal commitment and perceived goal progress, the participants were also asked to respond to the short version of the *Personal Goals Variables* scale (Brunstein, 1993; Italian version: Monzani et al., 2015). This self-report measure asked participants to rate the personal goal they had declared in terms of a number of goal variables by considering the previous month. In particular, this questionnaire measures *commitment to personal goals* (three items; sample item: “Even if it means considerable effort, I will do everything necessary to accomplish this goal”; *α* = .69), and *progress in goal achievement* (two items; sample item: “I have made a great deal of progress concerning this goal”; *α* = .60). The response format was a seven-point Likert scale ranging from “completely disagree” (1) to “completely agree” (4).

### Data Analysis

Considering the participants’ responses to the Brief COPE, CFA was performed with Mplus 6.0 (Muthén & Muthén, 1998-2010). Because the Brief COPE has a four-point response format and the preliminary analysis showed positively skewed distributions of the responses of several items, CFA with Maximum Likelihood with Robust Standard Errors (MLR) was performed. MLR is an estimation procedure robust to violations of normality. According to recommendations by Hu and Bentler (1998, 1999), the goodness of fit of the considered models was evaluated by several fit indices: the chi-square goodness of fit test (χ^2^), root mean square error of approximation (RMSEA), comparative fit index (CLI), Tucker-Lewis index (TLI), and standardized root mean square residual (SRMR). The fit of the models was first evaluated by the *χ*^2^ statistic. Due to the sensitivity of the *χ*^2^ statistic to sample size, other indices were used based on Hu and Bentler’s (1999) recommendations. Good model fit was indicated by a CFI and TLI above 0.95, an RMSEA below 0.06, and an SRMR below 0.80. In addition, the 90% confidence interval for RMSEA (CI_RMSEA_) was used to test the null hypothesis of poor model fit more precisely: in a well-fitting model, the upper limit should be below 0.08 and the lower limit should be close to zero. Lastly, the probability of close fit (PCLOSE) was also considered. This measure provided a one-sided test of the null hypothesis of close fit (i.e., RMSEA equals .05). Jöreskog and Sörbom (1996) suggested that the *p*-value for this test should be above .50. A reliability analysis was performed using the omega coefficient and the model-based approach to the reliability of a composite score (McDonald, 1999; Raykov & Shrout, 2002).

Subsequently, the best fitting model was expanded to assess the associations of coping responses with goal commitment and progress. Specifically, this model evaluated correlations among latent variables of coping responses and observed variables of goal commitment and goal progress. These latter two variables were computed as the mean of responses in their respective items.

## Results

The fit statistics for the five alternative models are shown in Table 2. Model A was the only model to exhibit good fit [*χ*^2^(259, *N* = 606) = 460.662; *p* = .000; RMSEA = 0.036; CI_RMSEA_ = 0.031 – 0.041; PCLOSE = 1.000; CFI= 0.965; TLI= 0.948; SRMR = 0.039]. The four remaining models had inadequate fit.

**Table 2:** Fit Statistics for the Five Assessed Models

Model	χ*^2^*	*df*	*p*	*RMSEA*	*CI_RMSEA_*	*PCLOSE*	*CFI*	*TLI*	*SRMR*
Model A	460.662	259	.000	0.036	0.031 – 0.041	1.000	0.965	0.948	0.039
Model B	1209.111	314	.000	0.069	0.065 – 0.073	.000	0.842	0.810	0.081
Model C	1795.309	301	.000	0.091	0.087 – 0.095	.000	0.737	0.670	0.117
Model D	2093.659	339	.000	0.093	0.089 – 0.096	.000	0.691	0.656	0.128
Model E	1475.733	301	.000	0.080	0.076 – 0.085	.000	0.784	0.748	0.100

*Note*. *χ^2^* = chi-square goodness of fit test; *df* = degree of freedom; *p* = probability; RMSEA = root mean square error of approximation; CI_RMSEA_ = 90% confidence interval for RMSEA; PCLOSE = probability of close fit; CFI = comparative fit index; TLI = Tucker-Lewis index; SRMR = standardized root mean square residual.

Table 3 reports the standardized factor loadings and omega coefficients of reliability for the identified coping responses of Model A. As shown, all items had significant and sizeable loadings on their respective factors. Specifically, all standardized factor loadings, ranging from .439 to .959, were above the cutoff of .40 for item-factor retention (Brown, 2006). Considering the cut-off of the reliability coefficient recommended by Nunnally and Bernstein (1994) and Kline (1999), all 14 coping dimensions exhibited excellent or good reliability.

**Table 3:** Standardized Factor Loadings and Omega Coefficient for the 14 Coping Response Dimensions

Coping Response	Item Number	Standardized Factor Loading (*SE*)	Omega
Self-distraction	1	.535 (.045)***	.849
	19	.822 (.054)***	
Active coping	2	.697 (.035)***	.744
	7	.858 (.023)***	
Denial	3	.640 (.043)***	.879
	8	.775 (.049)***	
Substance use	4	.843 (.074)***	.976
	11	.959 (.046)***	
Use of emotional support	5	.863 (.016)***	.950
	15	.911 (.013)***	
Use of instrumental support	10	.909 (.016)***	.930
	23	.867 (.015)***	
Behavioral disengagement	6	.709 (.041)***	.862
	16	.782 (.041)***	
Venting	9	.790 (.027)***	.863
	21	.781 (.029)***	
Positive reframing	12	.770 (.036)***	.822
	17	.720 (.036)***	
Planning	14	.816 (.026)***	.885
	25	.811 (.023)***	
Humor	18	.439 (.171)*	.837
	28	.714 (.067)***	
Acceptance	20	.549 (.058)***	.833
	24	.796 (.064)***	
Religion	22	.911 (.038)***	.945
	27	.897 (.040)***	
Self-blame	13	.759 (.048)***	.705
	26	.562 (.039)***	

*p < .05. **p < .01. ***p < .001.

Table 4 shows the estimated correlation matrix for the 14 latent dimensions of coping responses. Pearson’s correlation among the nine identified factors showed that all of the significant correlations ranged from .110 to .899 in terms of absolute value. Following guidelines by Cohen (1988), we interpreted correlations in Table 4 as measures of effect size. Specifically, correlations were considered weak (|.10| < r < |.29|), moderate (|.30| < r < |.49|), or strong (|.50| < r < |1|). There were strong positive correlations between Use of emotional support and Use of instrumental support, between Use of Emotional Support and Venting, and between Active coping and Planning. These three high correlations between first-order factors suggested the potential applicability of a second-order factor model with two higher-order factors: the first comprised Venting and the use of both emotional and instrumental support; the second factor comprised Active coping and Planning. The remaining dimensions (i.e., Self-distraction, Denial, Substance use, Behavioral disengagement, Positive reframing, Humor, Acceptance, Religion, and Self-blame) were introduced in this second-order model as first-order factors. Model A, a first-order factor model, is a less restricted baseline model to which the second-order model can be compared, given that the second-order model is nested within Model A (Yung, Thissen, & McLeod, 1999). Specifically, a chi-square difference test using the Satorra-Bentler scaled chi-square (Satorra, 1999) can be used to compare the fit of the first- and second-order models. Similar to Model A, the second-order factor model displayed a good fit [χ^2^(290, N = 606) = 624.425; *p* = .000; RMSEA = 0.044; CI_RMSEA_ = 0.039 – 0.048; PCLOSE = .986; CFI= 0.941; TLI= 0.923; SRMR = 0.049]. However, the chi-square difference test between the first- and the second-order model was significant (Δχ^2^(31) = 167.916, *p* = .000), indicating that Model A fits the data better than the second-order factor model.

**Table 4:** Estimated Correlation Matrix for the 14 Coping Dimensions

	1	2	3	4	5	6	7	8	9	10	11	12	13	14
**1**	-													
**2**	.110*	-												
**3**	.405***	.034	-											
**4**	.256***	-.002	.523***	-										
**5**	.432***	.392***	.379***	.227***	-									
**6**	.300***	.455***	.244***	.192***	.899***	-								
**7**	.422***	-.365***	.578***	.363***	.158**	.064	-							
**8**	.498***	.375***	.478***	.313***	.724***	.643***	.228***	-						
**9**	.422***	.462***	.212***	.185**	.500***	.448***	.077	.409***	-					
**10**	.105	.858***	-.029	-.023	.319***	.437***	-.329***	.360***	.443***	-				
**11**	.378***	.003	.352***	.313***	.271***	.231***	.400***	.399***	.453***	.087	-			
**12**	.270***	.533***	-.007	.042	.253***	.268***	.006	.394***	.476***	.501***	.274*	-		
**13**	.147**	.198***	.230***	.111	.310***	.297***	.058	.182***	.316***	.170***	.190*	.149**	-	
**14**	.456***	.368***	.218**	.195***	.419***	.395***	.208**	.501***	.591***	.561***	.479***	.355***	.190***	-

*Note*. 1 = Self-distraction; 2 = Active coping; 3 = Denial; 4 = Substance use; 5 = Use of emotional support; 6 = Use of instrumental support; 7 = Behavioral disengagement; 8 = Venting; 9 = Positive reframing; 10 = Planning; 11 = Humor; 12 = Acceptance; 13 = Religion; 14 = Self-blame.

*p < .05. **p < .01. ***p < .001.

Because Model A exhibited the best fit among the tested models, it was expanded to assess the associations of coping responses with goal commitment and progress. Specifically, this model evaluated correlations among latent variables of coping responses and observed variables of goal commitment and goal progress. These latter two variables were computed as the mean of responses in their respective items. This model exhibited good fit [*χ*^2^(287, *N* = 606) = 512.339; *p* = .000; RMSEA = 0.036; CI_RMSEA_ = 0.031 – 0.041; PCLOSE = 1.000; CFI= 0.963; TLI= 0.944; SRMR = 0.038]. Table 5 shows the correlations of 14 coping responses with both goal commitment and goal progress. There was a moderate and positive correlation between goal commitment and progress (*r* = .363, *p* < .001). As hypothesized, goal commitment was positively related to the coping response aimed at approaching both problems and emotions. Specifically, the highest correlations were with Active coping (*r* = .492, *p* < .001) and Planning (*r* = .488, *p* < .001). Regarding responses aimed at avoiding stressors, a negative and moderate correlation was found between commitment and Behavioral disengagement (*r =* -.352, *p* < .001).

**Table 5:** Correlations of Coping Responses With Goal Commitment and Progress

	Goal Commitment	Goal Progress
Self-distraction	.015	-.278***
Active coping	.492***	.256***
Denial	-.008	-.272***
Substance use	-.055	-.127**
Use of emotional support	.231***	.026
Use of instrumental support	.299***	.094*
Behavioral disengagement	-.352***	-.419***
Venting	.209***	-.099*
Positive reframing	.146**	.075
Planning	.488***	.208***
Humor	-.108	-.133*
Acceptance	.129*	-.045
Religion	.117**	.084
Self-blame	.193**	-.100

*p < .05. **p < .01. ***p < .001.

Similar patterns of correlations were obtained when considering goal progress, which was positively related to coping responses of approaching both problems and emotions. The highest positive correlations were between goal progress and Active coping (*r* = .256, *p* < .001) and Planning (*r* = .208, *p* < .001). Goal progress was negatively related to coping responses of avoiding both problems and emotions. Specifically, progress was inversely related to Behavioral disengagement (*r* = -.419, *p* <.001), Self-distraction (*r* = -.278, *p* <.001), Denial (*r* = -.272, *p* <.001), and Substance use (*r* = -.127, *p* <.01).

## Discussion

The main purpose of the present study was to evaluate the factor structure of the situational version of the Brief COPE and to evaluate its relationships with goal commitment and goal progress. The identification of the best factor structure of the Brief COPE is a necessary step in order to validly and reliably assess coping strategies in clinical practice and research context. Moreover, the chance of having a shared and common method for scoring coping strategies may permit to compare findings across different studies and thus better study this important aspect in psychological practice. The study represents the first attempt to compare and test the factor structure highlighted by previous research. The alternative models were tested by performing CFA with the MLR estimation method, which is assumed to be robust against violations of the assumption of normality. The results support the theoretically based 14-factor structure proposed by Carver (1997). Concerning the size of the standardized factor loadings, all items are good indicators of their respective factors. Moreover, considering the omega coefficient of reliability, the analysis finds acceptable internal consistency for all 14 coping dimensions. Thus, the empirical evidence supports the usefulness of the situational version of the Brief COPE for the valid and reliable assessment of 14 specific coping responses to stressors. Specifically, as theorized by Carver (1997), the 14 measured coping responses are Self-distraction, Active coping, Denial, Substance use, Use of emotional support, Use of instrumental support, Behavioral disengagement, Venting, Positive reframing, Planning, Humor, Acceptance, Religion, and Self-blame.

The correlation analysis provides important information on the reciprocal relationships among coping responses adopted to face stressors and threats. In general, most of the coping strategies are correlated with each other from weakly to strongly. In particular, the strong positive correlations among Use of emotional support, Use of instrumental support, and Venting and between Active coping and Planning suggest the possibility of advancing a second-order factor model that may better explain these relationships among first-order factors. However, the results of the second-order CFA demonstrate that the first-order 14-factor model fit the observed data better. According to the distinction suggested by Solberg Nes and Segerstrom (2006), Active coping, Use of instrumental support, and Planning could be categorized as approach problem coping responses. The Use of emotional support, Venting, Positive reframing, Acceptance, and Religion may be classified as approach emotion responses. Only Behavioral disengagement could be categorized as avoidance problem coping, and Self-distraction, Denial, and Substance use may classified as avoidance emotion coping responses. Humor and Self-blame could be considered emotion-focused strategies, but they are indistinguishable on the approach vs. avoidance dimension. Considering this theoretical distinction (Solberg Nes & Segerstrom, 2006), stronger links were observed between coping strategies belonging to the same category. However, significant correlations were also observed between coping responses in different classes. Future research should evaluate the applicability of the distinctions between the coping dimensions for approach problem, approach emotion, avoidance problem, and avoidance emotion.

Lastly, the results of our research showed significant relationships of the situational coping responses measured by the Brief COPE with goal commitment and goal progress. Consistent with the self-regulation theory, goal commitment was closely linked to the adoption of the approach coping responses of Active coping, Use of instrumental and emotional support, and Planning. When people are more committed to their goals, they are more likely to adopt strategies to actively address the stressor or related emotions. However, the relationship between coping dimensions and goal progress is more informative of the effectiveness and adaptiveness of this response to goal-related difficulties. As suggested by Lazarus (1991) and Weiss and Cropanzano (1996), effective coping responses are more likely to foster goal-directed behaviors and goal achievement. The results of our analysis demonstrated that coping responses of approaching problems are closely linked to goal performance. When people adopt Active coping and Planning responses to a specific goal-related difficulty, they are more likely to report progress toward a specific personal goal. In contrast, the coping response of avoiding both emotions and problems is more likely to interfere with personal goal pursuit. In particular, there are negative relationships among goal progress and Behavioral Disengagement, Self-distraction, Denial, and Substance use.

The current findings should be considered in light of three main limitations. First, the results regarding both the factor structure of the Brief COPE and its relations with goal commitment and progress are limited to the population considered in the study. Although the participants in the current study represent a large and heterogeneous community sample, these individuals do not necessarily represent the general population. Future studies should include representative and random samples of people of different provenances, cultures, and ethnicities. Second, cross-sectional data and correlational analyses were performed to evaluate the relationships of coping responses with goal progress and commitment. Future longitudinal research may also assess the causal effect of the adoption of specific coping responses on subsequent goal progress and performance and other variables, such as psychological adjustment and well-being. Lastly, the present study did not evaluate the role of other relevant aspects, such as socioeconomic status, dispositional coping style, dispositional optimism, causal attributions, and the controllability of stressors, on the adoption of coping responses when facing specific stressors and threats. Future research may evaluate their role in predicting the choice of coping strategy.

Despite these limitations, the results of the current study demonstrate the effectiveness of the 14-factor structure of the situational version of the Brief COPE. The situational version of this instrument asks people to report how they have coped with a specific stressor or threat. Our results confirm that the Brief COPE measures 14 different strategies of Self-distraction, Active coping, Denial, Substance use, Use of emotional support, Use of instrumental support, Behavioral disengagement, Venting, Positive reframing, Planning, Humor, Acceptance, Religion, and Self-blame. As a practical consequence, we advise that future research and clinical psychologists should use these 14 distinct scores of coping responses. Moreover, this study demonstrates that the Brief COPE is a useful self-report questionnaire to evaluate coping responses to specific difficulties and negative circumstances. Given its brevity and ease of administration, the Brief COPE could be conveniently introduced in both long research protocols and clinical assessment. Lastly, as previously suggested by Carver (1997), the Brief COPE may assume a dispositional format by slightly changing the phrasing of its instructions and items. This latter version could be developed to evaluate people’s dispositional coping styles by changing the instructions to ask people to report how they generally cope when they encounter a difficult or stressful situation. Similarly, the verb forms of items could be changed from the present perfect tense of the situational version to the present tense of the dispositional format.
